# Vascular PPARβ/δ Promotes Tumor Angiogenesis and Progression

**DOI:** 10.3390/cells8121623

**Published:** 2019-12-12

**Authors:** Kay-Dietrich Wagner, Siyue Du, Luc Martin, Nathalie Leccia, Jean-François Michiels, Nicole Wagner

**Affiliations:** 1 Université Côte d’Azur, CNRS, INSERM, iBV, 06107 Nice, France; kwagner@unice.fr (K.-D.W.); Siyue.DU@univ-cotedazur.fr (S.D.); Luc.MARTIN@univ-cotedazur.fr (L.M.); 2 Department of Pathology, CHU Nice, 06107 Nice, France; leccia.n@chu-nice.fr (N.L.); michiels.jf@chu-nice.fr (J.-F.M.)

**Keywords:** peroxisome-proliferator activated receptors, tumor angiogenesis, tumor progression, metastasis formation, endothelial cells, RNA sequencing

## Abstract

Peroxisome proliferator-activated receptors (PPARs) are nuclear receptors, which function as transcription factors. Among them, PPARβ/δ is highly expressed in endothelial cells. Pharmacological activation with PPARβ/δ agonists had been shown to increase their angiogenic properties. PPARβ/δ has been suggested to be involved in the regulation of the angiogenic switch in tumor progression. However, until now, it is not clear to what extent the expression of PPARβ/δ in tumor endothelium influences tumor progression and metastasis formation. We addressed this question using transgenic mice with an inducible conditional vascular-specific overexpression of PPARβ/δ. Following specific over-expression of PPARβ/δ in endothelial cells, we induced syngenic tumors. We observed an enhanced tumor growth, a higher vessel density, and enhanced metastasis formation in the tumors of animals with vessel-specific overexpression of PPARβ/δ. In order to identify molecular downstream targets of PPARβ/δ in the tumor endothelium, we sorted endothelial cells from the tumors and performed RNA sequencing. We identified platelet-derived growth factor receptor beta (Pdgfrb), platelet-derived growth factor subunit B (Pdgfb), and the tyrosinkinase KIT (c-Kit) as new PPARβ/δ -dependent molecules. We show here that PPARβ/δ activation, regardless of its action on different cancer cell types, leads to a higher tumor vascularization which favors tumor growth and metastasis formation.

## 1. Introduction

Peroxisome proliferator-activated receptors (PPARs) are nuclear receptors. They function as ligand activated transcription factors. They exist in three isoforms, PPARα, PPARδ (formerly PPARβ), and PPARγ. For all PPARs, lipids are endogenous ligands, linking them directly to metabolism. PPARs are considered as important transcriptional regulators of genes involved in lipid metabolism and cardiac energy production [1]. PPARs form heterodimers with retinoic X receptors, and, upon ligand binding, modulate gene expression of downstream target genes dependent on the presence of co-repressors or co-activators. This results in cell-type specific complex regulations of proliferation, differentiation, and cell survival. Specific synthetic agonists for all PPARs are available. PPARα and PPARγ agonists are already in clinical use for the treatment of hyperlipidemia and type 2 diabetes, respectively. More recently, PPARβ/δ activation came into focus as an interesting novel approach for the treatment of metabolic syndrome and associated cardiovascular diseases.

Agonists of PPARβ/δ improve insulin sensitivity in both, murine models and in humans. Thus, they are considered a potential target in the treatment of obesity and obesity associated disorders [2]. Metabolic syndrome is regarded as a high-risk state for cancer. Targeting of PPARβ/δ was suggested for the treatment of metabolic syndrome (reviewed in [3]), but the resulting consequences for cancer risk are less clear. The function of PPARβ/δ in different types of cancer is highly controversial at present. This might result from the different experimental models used and also from the varying contribution of PPARβ/δ to endothelial cell proliferation, inflammation, and tumor cell proliferation, differentiation, or apoptosis (reviewed in [4]). As all these processes are critically involved in cancer growth; different approaches could give rise to opposing results. We recently reported that PPARβ/δ inhibits melanoma cell proliferation through the transcriptional repression of the Wilms’ tumor suppressor WT1 [5]. In contrast, we found that PPARβ/δ increases liposarcoma cell proliferation through the direct repression of the adipose tissue secretory factor leptin [6].

PPARβ/δ, unlike PPARα or PPARγ, appears to be a predominantly pro-angiogenic signaling molecule. PPARβ/δ is expressed in endothelial cells and pharmacological activation of endothelial cells with PPARβ/δ agonists had been shown to increase the angiogenic properties of these cells [7]. PPARβ/δ is also involved in physiological angiogenesis. As we and others showed, treatment with the PPARβ/δ agonists GW0742 and GW501516 induced an exercise-like phenotype in the heart. Both agonists induced a surprisingly rapid (after 24 h) remodeling of mouse hearts [8] and skeletal muscle [9] by increasing micro-vessel densities. However, until now it was not clear whether either the increase of the cardiac vasculature drives the myocardial hypertrophy or the enhanced cardiac angiogenesis might be a potential indirect effect of cardiomyocyte-specific PPARβ/δ activation. In a recent work, we addressed this question through the generation of transgenic mice with an inducible conditional vascular-specific overexpression of PPARβ/δ and analyzed the normal cardiac phenotype and function as well as morphology and function under chronic ischemic heart disease conditions. We showed that inducible vessel-specific overexpression of PPARβ/δ results in a rapid induction of angiogenesis, cardiac hypertrophy, and impairment of cardiac function. Additionally, we demonstrated that after myocardial infarction, despite the higher collateral vessel formation, the animals with vascular- specific PPARβ/δ overexpression display bigger infarct lesions, higher cardiac fibrosis, and further reduced cardiac function. This points to a more careful view about the potential benefits of PPARβ/δ agonists in the treatment of cardiovascular diseases as the proper balance between cardiomyocytic and vascular PPARβ/δ seems to be crucial for cardiac health, especially under ischemic conditions [10]. Although the function of PPARβ/δ in lipid and glucose metabolism, and the remodeling of skeletal and cardiac muscle is well established, its role in tumor angiogenesis and cancer progression is unclear or at least partially controversial. It is of great importance to clarify the impact of PPARβ/δ activation in the vasculature particularly in cancer, as caution may be required when testing PPARβ/δ activation where more angiogenesis may play important pathological roles. The question of the safety of a potential use of PPAR β/δ modulation in clinical studies therefore needs to be answered urgently.

In human pancreatic tumors, PPARβ/δ expression strongly correlated with advanced tumor stage and increased risk of tumor recurrence and distant metastasis [11]. PPARβ/δ has therefore been suggested to be involved in the regulation of the angiogenic switch in tumor progression. However, until now it is not clear to what extent the expression of PPARβ/δ in the tumor endothelium influences tumor progression and metastasis formation. We determine in this study the in vivo relevance of PPARβ/δ for tumor vessel formation and cancer growth using modern mouse genetic approaches. We first tested the effects of the PPARβ/δ agonist GW0742 on tumor cell proliferation in vitro and in vivo. Although the PPARβ/δ agonist inhibited cancer cell proliferation in vitro, we could observe enhanced tumor growth upon treatment in vivo. Tumors of animals treated with the PPARβ/δ agonist displayed higher vessel densities and formed more metastases compared to controls. We next generated an inducible conditional endothelial cell-specific over-expression mouse model for PPARβ/δ to analyze the effects on tumor angiogenesis and progression. We show here that tumor angiogenesis and cancer growth as well as metastasis formation are increased upon endothelial cell-specific upregulation of PPARβ/δ. Furthermore, we identify platelet-derived growth factor receptor beta (Pdgfrb), platelet-derived growth factor subunit B (Pdgfb), and the tyrosinkinase KIT (c-Kit) as new PPARβ/δ -dependent molecules involved in tumor vessel formation based on RNA sequencing and subsequent verification applying a variety of molecular biology approaches.

## 2. Materials and Methods

### 2.1. Animals

All animal work was conducted according to national and international guidelines and was approved by the local ethics committee (Comité Institutionnel d‘Éthique Pour l‘Animal de Laboratoire Azur (Ciepal), agreement number: *PEA-NCE/2013/334).* PPARβ/δ-flox^+/−^ [12] and Tie2-CreERT2 [13] animals were crossed to generate Tie2-CreERT2;PPARβ/δ-flox^+/−^ mice, further referred to as Tie2-CreERT2;PPARβ/δ. The Tie2-CreERT2-line was back-crossed four times onto C57BL/6J. Age- and sex-matched Tie2-CreERT2;PPARβ/δ animals were injected for one week intraperitoneally either with sunflower oil (vehicle) or Tamoxifen dissolved in sunflower oil in a dose of 33  mg/kg per day [10,14,15]. Tie2-CreERT2 animals injected with Tamoxifen served as additional controls. One week after the last Tamoxifen or vehicle treatment, 1 × 10^6^ LLC1 tumor cells were injected subcutaneously. Tumors and organs were collected after three weeks. For treatment with the PPARβ/δ agonist, ten-week-old male C57BL/6J (Janvier, France) mice were subcutaneously injected with 1 × 10^6^ LLC1 tumor cells. GW0742 (Selleckchem, Houston, TX, USA) dissolved in DMSO was then subcutaneously injected at 1 mg/kg once every second day (100 µL). Controls received 100 µL DMSO injections [8].

### 2.2. Cell Culture

Human umbilical vein endothelial cells (HUVEC) were purchased from PromoCell (Heidelberg, Germany) and grown in endothelial cell growth medium (PromoCell) supplemented with gentamycin (50 µg mL^−1^) and amphotericin B (50 ng mL^−1^). For all experiments, we used HUVECs pooled from up to four donors, which did not exceed passage 4. Human embryonic kidney (HEK) 293 cells (ATCC CRL-1573) were grown in DMEM medium (Invitrogen, Cergy Pontoise, France) supplemented with 10% fetal calf serum (FCS), 100 IU mL^−1^ penicillin, and 100 µg mL^−1^ streptomycin (Invitrogen, Cergy Pontoise, France). C166 mouse endothelial cells (accession number CRL-2581) and LLC1 mouse lung cancer cells (accession number CRL-1642) were grown in DMEM medium (Invitrogen, Cergy Pontoise, France). Media were supplemented with 10% fetal calf serum (FCS), 100 IU mL^−1^ penicillin and 100 µg mL^−1^ streptomycin. As positive control for apoptosis assays, LLC1 mouse lung cancer cells were treated with 100 nmol/L Staurosporine (Sigma, St. Louis, MO, USA) overnight. For RNA isolation and quantitative RT-PCR experiments, HUVEC and LLC1 cells were maintained for 48 h (HUVEC) or 24 h (LLC1) in medium in the presence of GW0742 (Selleckchem, Houston, TX, USA) or GSK3787 (Selleckchem) dissolved in dimethyl sulfoxide (DMSO) at concentrations of 1 µmol/L. Controls were treated with vehicle (0.1% DMSO) only [6,16].

### 2.3. Detection of Cell Proliferation 

After incubation for 24 h (LLC1 cells) or 48 h (HUVECs) with DMSO, GW0742, or GSK3787, bromodeoxyuridine was added and the cells incubated for 3 h. Afterwards, BrdU incorporation was measured spectrophotometrically according to manufacturer’s instructions (Millipore, Molsheim, France). Alternatively, cells were labeled with a mouse monoclonal proliferating cell nuclear antigen (PCNA) antibody (PC-10, Santa Cruz Biotechnology, Heidelberg, Germany) and 4′,6-diamidino-2-phenylindole (DAPI) counterstain (Vector Laboratories, Burlingame, CA, USA). PCNA-positive cells in five random optical fields from six independent experiments each were counted at 400× magnification. 

### 2.4. Apoptosis Assays 

Apoptotic cells were detected by Terminal deoxynucleotidyl transferase dUTP nick end labeling (TUNEL) staining of HUVECs, 48 h after treatment with DMSO, GW0742, or GSK3787 using the In Situ Cell Death Detection Kit (Roche Molecular Biochemicals, Meylan, France) according to the manufacturer’s instructions. LLC1 cells were incubated with APC-conjugated annexin V (Roche, Meylan, France) and counterstained with propidium iodide to distinguish necrotic from apoptotic cell death. LLC1 cells treated with 100 nmol/L Staurosporine (Sigma, St. Louis, MO, USA) overnight served as positive controls.

### 2.5. Immunofluorescence Assays 

Cells were fixed for 10 min on ice with 4% paraformaldehyde in phosphate-buffered saline (PBS). After PBS washes, cells were incubated for 1 h at room temperature in blocking solution (1% Triton X-100, 1%BSA, 5% donkey serum in PBS). Cells were then immuno-stained overnight at 4 °C in blocking solution containing the following primary antibodies: rabbit polyclonal anti PPARβ/δ (ThermoFisher Scientific, Nimes, France, 1:200) and mouse monoclonal PDGFRB (ThermoFisher Scientific, 1:300), or goat polyclonal PDGFB antibody (Abcam, Cambridge, UK, 1:50), or mouse monoclonal anti c-Kit (Abcam, 1:500). After three washes with PBS/0.1% Triton X-100, slides were incubated for 1 h 30 min at room temperature with Dylight 488 donkey anti-mouse or Dylight 488 donkey anti-goat and Dylight 594 donkey anti-rabbit secondary antibodies in PBS containing 0.5% Triton X-100, 1%BSA, 2.5% donkey serum. Slides were mounted with Vectashield with DAPI (Vector Laboratories, Burlingame, USA). Slides were viewed under an epifluorescence microscope (DMLB, Leica, Germany) connected to a digital camera (Spot RT Slider, Diagnostic Instruments, Scotland). 

### 2.6. Endothelial Cell Isolation

Mouse tumor endothelial cells (EC) were isolated from Tie2-CreERT2;PPARβ/δ mice treated with Tamoxifen or vehicle as previously described [10,17,18]. Tie2-CreERT2 animals injected with Tamoxifen served as an additional control. Briefly, tumor tissues were cut into small fragments and digested with 1 mg/mL collagenase A and 100 IU/mL type I DNase (Roche Diagnostics, Meylan, France) for 45 min at 37 °C. ECs were then purified from the cell suspension using CD31 MicroBeads followed by magnetic separation in LS columns (Miltenyi Biotec SAS, Paris, France). 

### 2.7. RT-PCR and Quantitative RT-PCR 

Total RNA was isolated using the Trizol reagent (Invitrogen). First-strand cDNA synthesis was performed with 0.5 µg of total RNA using the Thermo Scientific Maxima First Strand cDNA synthesis kit (Thermo Scientific). The reaction product was diluted to 200 µL and 1 µL of the diluted reaction product was taken for real time RT-PCR amplification (StepOne plus, Applied Biosystems) using the SYBR^®^ Select Master Mix (Applied Biosystems). Expression of each gene was normalized to the respective arithmetic means of *Gapdh*, *Actnb*, and *Rplp0* expression.

Primer sequences for mouse genes are given in Table 1 and for human genes in Table 2.

### 2.8. mRNA Sequencing

For sequencing, RNAs from tumor sorted endothelial cells from Tie2-CreERT2;PPARβ/δ mice treated with Tamoxifen or vehicle were used (*n* = 4 each). RNA sequencing and data analysis was performed by Novogene, Beijing, China. Briefly, RNA quality was monitored on 1% agarose gels. RNA purity was checked using a NanoPhotometer. RNA concentration was measured using the Qubit^®^ RNA Assay Kit in a Qubit^®^ 2.0 Flurometer (Life Technologies, CA, USA). RNA integrity was assessed using the RNA Nano 6000 Assay Kit of the Bioanalyzer 2100 system (Agilent Technologies, CA, USA). A total amount of 1 μg RNA per sample was used as input material for the RNA sample preparations. Sequencing libraries were generated using NEBNext^®^ UltraTM RNA Library Prep Kit for Illumina^®^ (NEB, USA) following manufacturer’s instructions. Library quality was assessed on the Agilent Bioanalyzer 2100 system. Library preparations were sequenced on an Illumina Hiseq platform and 125 bp/150 bp paired-end reads were generated. Clean reads were obtained by removing reads containing adapter, reads containing ploy-N and low quality reads from raw data. HTSeq v0.6.1 was used to count the reads numbers mapped to each gene. Fragments per kilobase of transcript sequence per millions base pairs sequenced (FPKM) of each gene was calculated based on the length of the gene and reads count mapped to this gene. Differential expression analysis of the two groups was performed using the DESeq R package (1.18.0). Resulting *p*-values were adjusted using the Benjamini and Hochberg’s approach. Genes with an adjusted *p*-value < 0.05 were assigned as differentially expressed. The RNA sequencing data have been deposited in NCBI’s Gene Expression Omnibus [19,20] and are accessible through GEO Series accession number GSE140513 (https://www.ncbi.nlm.nih.gov/geo/query/acc.cgi?acc=GSE140513).

### 2.9. Bioinformatics 

The maximum scoring subnetwork was calculated with the runFastHeinz function from the R BioNet package [21]. The *p*-values obtained from the differential expression were assigned to each node (gene) of the networks. The following networks were analyzed: FULL-Mouse (18 January, 2019) from signor database (http://signor.uniroma2.it/), HumanCyc metabolic pathways (http://humancyc.org/), NCI PID, Complete Interactions (http://www.cancer.gov), Biogrid (https://thebiogrid.org/), HCOP Mouse PCNet [22], ConsensusPathDB (http://cpdb.molgen.mpg.de/). All networks were downloaded from http://www.ndexbio.org [23]. Sub-network visualizations and analyses were done with Cytoscape [24] and pathway cluster analysis at http://impala.molgen.mpg.de/ [25]. Prediction of PPAR, responsive elements in differentially expressed genes was done using the oPOSSUM3 software at http://opossum.cisreg.ca/oPOSSUM3/ [26]. 

### 2.10. Tissue Samples and Immunohistology

The study adheres to the principles of the Declaration of Helsinki and to Title 45 of the U.S. Code of Federal Regulations (Part 46, Protection of Human Subjects). Paraffin-embedded samples, cut at 3µm, were used for immunohistochemical detection of PPARβ/δ, CD31, and PCNA. In total, 35 paraffin-embedded human tumor samples (7 liver carcinomas, 7 melanomas, 7 pancreas carcinomas, 7 ovary carcinomas, and 7 prostate cancers) were used for this study. For immunofluorescence double-labeling of human tumor samples, anti-CD31 mouse monoclonal antibody from Dako (Trappes, France, clone JC70A) was combined with the anti-PPARβ/δ antibody (ThermoFisher Scientific), using Dylight 488 donkey anti rabbit and Dylight 594 donkey anti mouse secondary antibodies. Negative controls were obtained by omission of first antibodies. Images were taken using a confocal ZEISS LSM Exciter microscope (Zeiss, Jena, Germany).

Mouse tissue sections were in routine stained with hematoxylin-eosin. After heat-mediated antigen retrieval and quenching of endogenous peroxidase activity, PPARβ/δ (ThermoFisher Scientific) or CD31 (rabbit polyclonal, 1:50, Abcam) were detected using the EnVisionTM Peroxidase/DAB Detection System from Dako. Negative controls were obtained by incubation of samples with a rabbit IgG Control (Abcam). Sections were counterstained with hematoxylin (Dako) and analyzed by two independent investigators, one of them an experienced pathologist. Slides were viewed under an epifluorescence microscope (DMLB, Leica, Germany) connected to a digital camera (Spot RT Slider, Diagnostic Instruments, Scotland).

### 2.11. Cloning and Transient Transfection Experiments

The PDGFRβ promoter was amplified from HUVEC genomic DNA using the following primers: 5′-TAGGTACCAAAGACTTAGCGGCGCAGAG-3′ (forward, position -1766), 5′-GTGAGATCTCTGCCCTCTCCCAGTTATCAG-3′ (backward, position +374) and cloned into the KpnI/BglII restriction sites of pGl3 basic [17]. The Pdgfb promoter was amplified from mouse genomic DNA using the following primers: 5′-CGGGGTACCATCAGTACCACCTCATCCA-3′ (forward, position -1199), 5′-CCCAAGCTTCTCGGGTCAGTCTGTCTA-3′ (backward, position +98) and cloned into the KpnI/HindIII restriction sites of pGl3 basic. The c-Kit promoter was a kind gift of C. Nishiyama [27]. As vector backbone, pGl3 basic (Promega) was used for all constructs. The putative PPARβ/δ responsive elements sites were deleted from the PDGFRB promoter construct using the Quik Change II site directed mutagenesis kit (Stratagene, Agilent Technologies, Massy, France) with the following oligonucleotides: PPRE1: 5′-ATATCCAATCTGTGCTGGAATCACATTCCCTCTCTGTG-3′; antisense: reverse complement; PPRE2: 5′-TCATGTGTCTCATGAGACCTAGTTCTGCCATTGCTGC-3′; antisense: reverse complement; PPRE3: 5′-ATATCCAATCTGTGCTGGAATCACATTCCCTCTCTGTG-3′; antisense: reverse complement. The putative PPARβ/δ responsive elements sites were deleted from the Pdgfb promoter construct using the Quik Change II site directed mutagenesis kit (Stratagene) with the following oligonucleotides: PPRE1: 5′-GTGGGTGGGTAGCGAACTGGGTGGGG-3′; antisense: reverse complement; PPRE2: 5′-GACAAGCAAGGAGAGGTGTAGCTGAAGGGTTC-3′; antisense: reverse complement; PPRE3: 5′-GAAGGAAAGTGACGTGCCCAAGATTTAATTAGACTCAATGGAATC-3′; antisense: reverse complement. For deletion of putative PPARβ/δ responsive element sites in the c-kit promoter we used oligonucleotides PPRE1: 5′-TACCAACAGGAACAGAAATAAATGTTCCTAATCCCTTCGCC-3′; antisense: reverse complement; PPRE2: 5′-TGGGCTCGGTCTTTTACGGGTGCCACGATC-3′; antisense: reverse complement.

HEK-293 and C166 cells at approximately 60% confluence were transfected using Fugene 6 reagent (Roche, Meylan, France). Reporter constructs (full promoter sequences and sequences with deletions of the PPARβ/δ responsive elements sites) were co-transfected with a cytomegalovirus (CMV)-driven galactosidase plasmid, and the PPARβ/δ expression construct, and assayed for luciferase- and galactosidase activity (*n* = 12 each).

### 2.12. Chromatin Immunoprecipitation Assay

Chromatin immunoprecipitation (ChIP) assay was performed on HEK293 (human PPREs) or C166 (mouse PPREs) cells using manufacturer’s instructions (Millipore) as described [15,17,18]. One microgram of the following antibodies each were used: PPARβ/δ rabbit polyclonal, (H-74, Santa Cruz Biotechnology), PPARβ/δ goat polyclonal (K-20, Santa Cruz Biotechnology), Acetyl-Histone H3 (06-599, Upstate). Omission of primary antibodies served as a negative control for the PPARβ/δ antibodies and dilutions of the input sample as positive control. PCR products were electrophoresed on 4% agarose gels. Alternatively, samples were used in quantitative PCRs (*n* = 3 each). Fold enrichment was calculated from CT values relative to the input signal of each experiment set to 100%. The following oligonucleotides were used for PCR amplification of the CHIP products: PDGFRβPPRE1: 5′-GGTAAGCCCACTCTATATGCCCTTCTAA-3′ (forward); 5′-CCAGTTACAGACTCCTAGCCCTCAG-3′ (reverse); PDGFRβPPRE2: 5′-GGTCAGATGACTTGTGTCTCTTCCA-3′ (forward); 5′-CTTACGCAGCAATGGCAGAGC-3′ (reverse); PDGFRβPPRE3: 5′-GGGCTTTGAGACGTGAAAAGGA-3′ (forward); 5′-ATTGGCACAGAGAGGGAATGTG-3′ (reverse); PDGFRβUTR: 5′-CAGGTCCAGGTGAGTCAT-3′ (forward); 5′-CCTCTTCCTCTTCCTCTTCT-3′ (reverse); PdgfbPPRE1: 5′-AGGTGTTAACTGTGAGAGTG-3′ (forward); 5′-TGTTTACTACCCCTCTCTGC-3′ (reverse); PdgfbPPRE2: 5′-TCAACAGACTCAAATTCAGC-3′ (forward); 5′-CTCTAAACCCACAGCCAG-3′ (reverse); PdgfbPPRE3: 5′-ATCACAGAAGGAAAGTGACG-3′ (forward); 5′-AGAACCAGACATCTGCAAC-3′ (reverse); PdgfbUTR: 5′-GCTGGAGATAACCTTGGCTAAG -3′ (forward); 5′-GTTGGGACTCAGGATAGACTCA-3′ (reverse); c-kitPPRE1: 5′-TGGAGAAACTGAGCATGAAA-3′ (forward); 5′-TTCTGTTCCTGTTGGTAGAG-3′ (reverse); c-kitPPRE2: 5′-CTCTACCAACAGGAACAGAA-3′ (forward); 5′-CTTATGGTGGAGGTGTTACTA-3′ (reverse); c-kitUTR: 5′-CGATCTCATGTGGTCCAA-3′ (forward); 5′-CGCCTTGTTCATTACTACTG-3′ (reverse).

### 2.13. Statistical Analysis

Data are expressed as mean ± SEM. Student’s t-tests (Instat, GraphPad) were performed to determine statistical significance. A *p*-value of less than 0.05 was considered significant.

## 3. Results and Discussion

### 3.1. The PPARβ/δ Agonist GW0742 Decreases LLC1 Lewis Lung Cancer Cell Proliferation In Vitro

**Figure 1:**
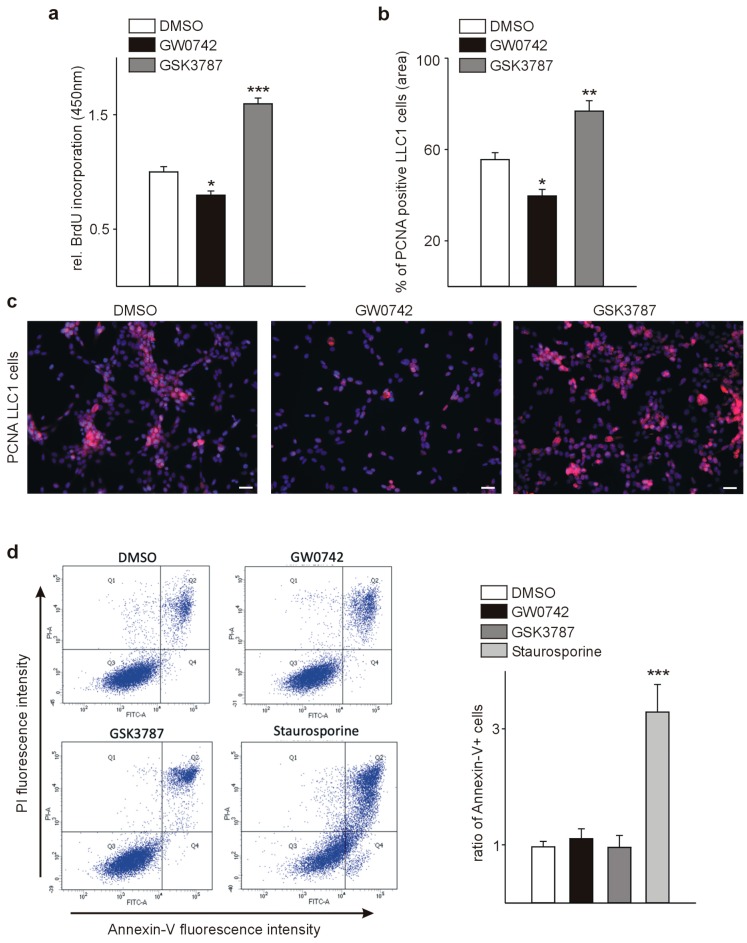
The PPARβ/δ agonist GW0742 decreases and the PPARβ/δ antagonist GSK3787 increases LLC1 proliferation. (**a**) Quantification of bromodeoxyuridine (BrdU) incorporation assays (*n* = 8) (**b**) and the proportion of proliferating cell nuclear antigen (PCNA)-positive cells (**c**) as measures of proliferation (*n* = 4). (**d**) fluorescence-activated cell sorting (FACS) analysis of Annexin V/propidiumiodide labeled LLC1 cells after modulation of PPARβ/δ to measure apoptosis. Staurosporin-treated cells were used as positive control (*n* = 4). Scale bars indicate 50µm (c). Data are mean ± SEM. ^∗^ *p* < 0.05, ^∗∗^ *p* < 0.01, ^∗∗∗^ *p* < 0.001.

As a prerequisite before in vivo treatment of tumor-bearing mice with a PPARβ/δ agonist, we investigated the in vitro effects on syngenic tumor cells, which we aimed to inject into animals. LLC1 cells were treated for 24 h with the PPARβ/δ agonist GW0742 or the PPARβ/δ antagonist GSK3787 at a concentration of 1 µmol/L. Thereafter, we performed bromodeoxyuridine (BrdU) incorporation assays and immunostaining for proliferating cell nuclear antigen (PCNA), with subsequent quantification of PCNA-positive cells relative to the total cell number. GW0742 decreased BrdU incorporation and the fraction of PCNA-positive cells significantly, GSK3787 had the opposite effects (Figure 1a–c). These findings are in line with the effects of PPARβ/δ modulation on the proliferation of melanoma cells reported by our group [5] and recently confirmed in a study where a PPARβ/δ antagonist enhanced melanoma progression [28]. To determine whether apoptosis might contribute to the observed effects on cell growth upon GW0742 treatment, we used Annexin V/propidium iodide (PI) labeling to detect apoptotic events followed by fluorescence-activated cell sorting (FACS) analysis. Apoptosis of LLC1 cells was neither influenced by the PPARβ/δ agonist GW0742, nor the antagonist GSK3787 (Figure 1d).

As mentioned before, the role of PPARβ/δ in cancer is highly controversial. Concerning lung cancer, two studies reported an increased expression of PPARβ/δ in human non-small cell lung cancer (NSCLC) compared to normal lung and observed in NSCLC cell lines that PPARβ/δ activation promoted proliferation [29,30]. However, an earlier report observed an inhibition of proliferation in a NSCLC cell line upon PPARβ/δ activation [31] and finally, neither growth promoting nor inhibiting effects were observed in another study [32]. Thus, it is difficult to draw a definitive conclusion regarding the biological effect of PPARβ/δ in human lung cancer. There is also a high variability in the kind and concentration of PPARβ/δ agonists used, which might further contribute to these conflicting results. Nevertheless, the aim of our study was not to determine the effects of PPARβ/δ modulation in one cancer cell line, but to clarify the in vivo relevance of PPARβ/δ for tumor vessel formation and cancer growth, which necessitated to distinguish between direct effects on tumor cells and effects on the tumor microenvironment, especially tumor vessels.

### 3.2. The PPARβ/δ Agonist GW0742 Increases LLC1 Lewis Lung Cancer Progression In Vivo

**Figure 2:**
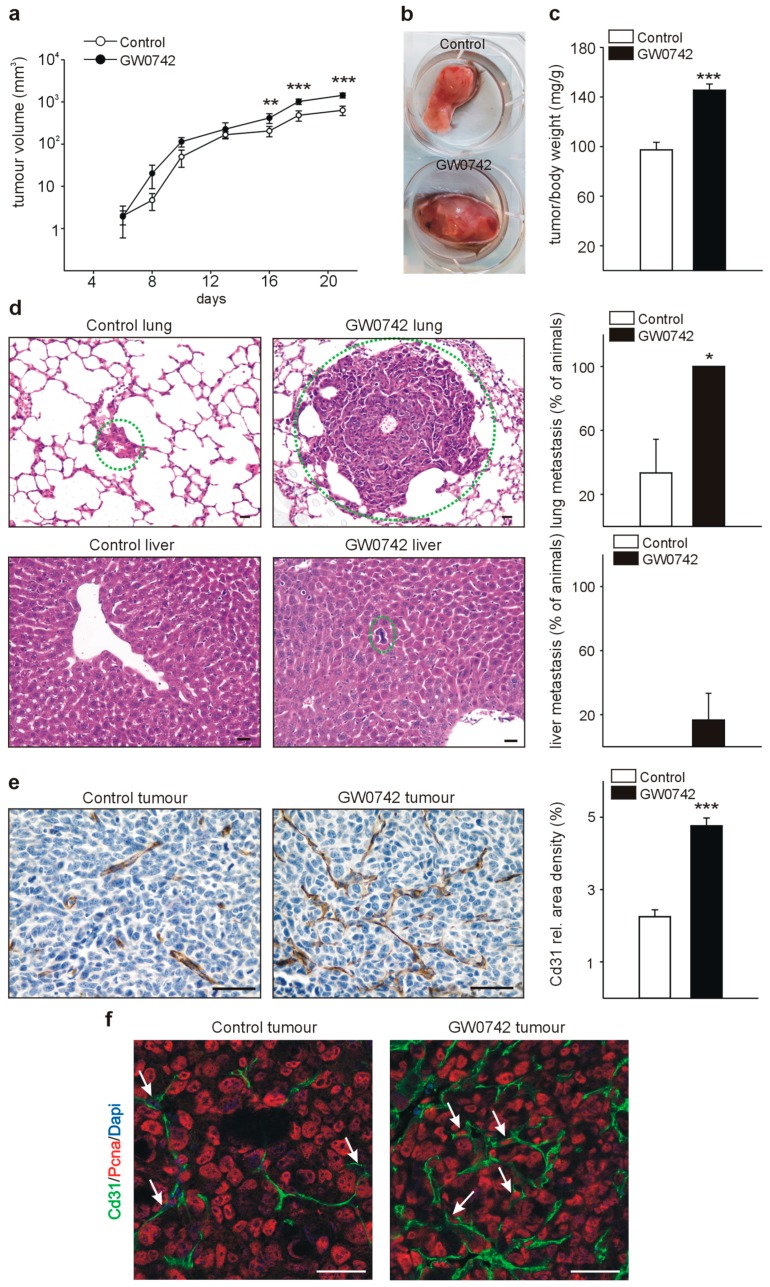
Treatment with the PPARβ/δ agonist GW0742 increases LLC1 growth, angiogenesis, and metastases formation in vivo. (**a**) LLC1 tumor growth curves in animals treated with GW0742 and respective controls (DMSO) (*n* = 6). Tumor volume data were calculated from Caliper measurements as described [15]. (**b**) LLC1 tumor in mice treated with GW0742 and respective control (DMSO). (**c**) Quantification of tumor/body weights in GW0742 and DMSO treated mice. (**d**) Representative photomicrographs of lung (upper panel) and liver (lower panel) metastases in the two groups of mice. Metastases are indicated with a dotted green circle. Note that in the animals with vehicle (DMSO), no liver metastasis was detectable. Graphs on the right show quantification of the percentage of animals with lung (upper graph) or liver (lower graph) metastasis from LLC1 tumors. (**e**) Cd31 immunostaining in LLC1 tumors and quantification of Cd31 signal area densities. (**f**) PCNA/Cd31 double-labeling of LLC1 tumors. Scale bars indicate 50 µm. Data are mean ± SEM. ^∗^ *p* < 0.05, ^∗∗^ *p* < 0.01, ^∗∗∗^ *p* < 0.001.

We investigated the effect of the PPARβ/δ agonist GW0742 on tumor growth by subcutaneous implantation of LLC1 tumor cells and subsequent injections of GW0742. Control animals received DMSO injections in the same frequency. Tumor growth was comparable between the two groups in the first days and started to increase in the animals receiving the PPARβ/δ agonist in the second week (Figure 2a). After three weeks, we determined tumor-to-body weight ratios in both groups and observed significantly higher tumor weights in GW0742 treated animals (Figure 2b,c). Next, we analyzed whether treatment with a PPARβ/δ agonist might modify metastasis occurrence. We investigated serial lung and liver sections of both groups for metastases formation. All animals, which had received the PPARβ/δ agonist displayed lung metastases formation, whereas only 30% of the animals in the control group had metastases occurrence in the lung. Lung metastases were not only more frequent in the treated group, but also of a bigger size than in controls. Liver metastases could be observed in 16% of the GW0742 treated mice, whereas none of the control animals had liver metastases (Figure 2d). Vessel density in tumors was determined by immunostaining for Cd31. Strikingly, in tumors of GW0742 treated mice, vessel densities were more than doubled compared to controls (Figure 2e). Double-staining for Cd31 and PCNA suggested a higher number of PCNA-positive endothelial cells in the tumors of PPARβ/δ agonist treated animals (Figure 2f).

Although the PPARβ/δ agonist GW0742 inhibited tumor cell proliferation in vitro, we could observe a strikingly different situation in vivo, where tumor growth and metastasis formation were enhanced. Tumor vascularization was strongly increased, which is in line with previous studies reporting a rapid boost of vascularization after pharmacological PPARβ/δ activation [8,9,10]. The increased tumor vascularization upon PPARβ/δ activation seems therefore to be sufficient to dominate over the anti-proliferative effect of the PPARβ/δ agonist on tumor cells. Increasing neovascularization is not only required for further expansion of the tumor-cell population, but also correlates with a rising rate of metastasis. In agreement with our findings, a recent study observed a marked inhibition of tumor angiogenesis and growth in PPARβ/δ^−/−^ mouse models of subcutaneous Lewis lung carcinoma and B16 melanoma, with occurrence of immature, leaky microvascular structures [33]. However, another group demonstrated enhanced extravasation of B16 melanoma cells in PPARβ/δ^−/−^ mice using an experimental model of metastases formation by injecting the tumor cells in the tail vein of animals, resulting in enhanced pulmonary metastases as compared to wildtype animals [28]. In contrast to the patho-physiologically occurring spontaneous metastasis formation, this assay does not require the formation of functional blood vessels necessary for the propagation of the primary tumor cells to other organs. The enhanced pulmonary metastases formation observed in the PPARβ/δ^−/−^ animals could therefore simply be due to the presence of non-functional leaky microvessels [33], facilitating the anchorage of the tumor cells in the lung.

### 3.3. PPARβ/δ Is Expressed in the Vasculature of Human Tumors and PPARβ/δ Modulation Impacts Proliferation and Expression of Pro-Angiogenic Factors in Human Endothelial Cells

**Figure 3:**
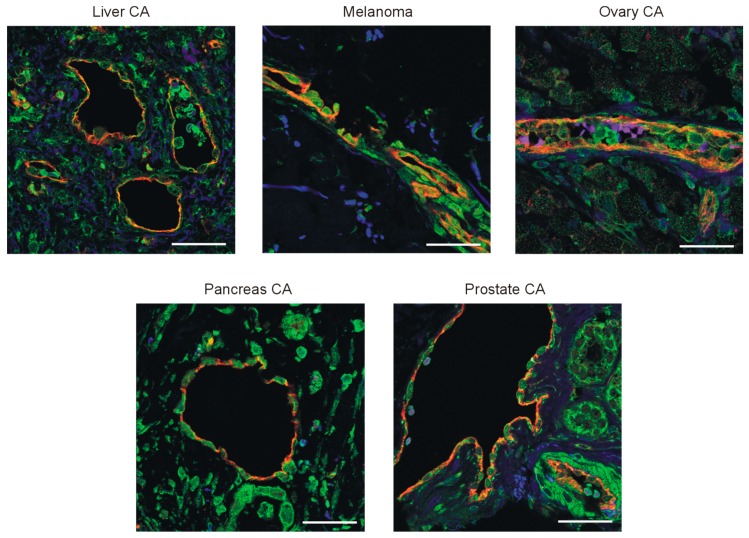
PPARβ/δ is expressed in human tumor vessels. Double labeling of PPARβ/δ (green) and cell adhesion molecule-1 (CD31) (red) in human tumor samples. Nuclei were counterstained with DAPI. Scale bars indicate 50 µm.

To confirm a relevance for human pathophysiology, we investigated human tumor samples (liver, pancreas, ovary, and prostate carcinoma, as well as melanoma) for expression of PPARβ/δ in the tumor vasculature. Confocal microscopy of tumor samples labeled for PPARβ/δ and CD31 confirmed co-localization of PPARβ/δ in endothelial cells (Figure 3). Furthermore, PPARβ/δ could be detected in tumor cells at various degrees in different tumor types.

**Figure 4:**
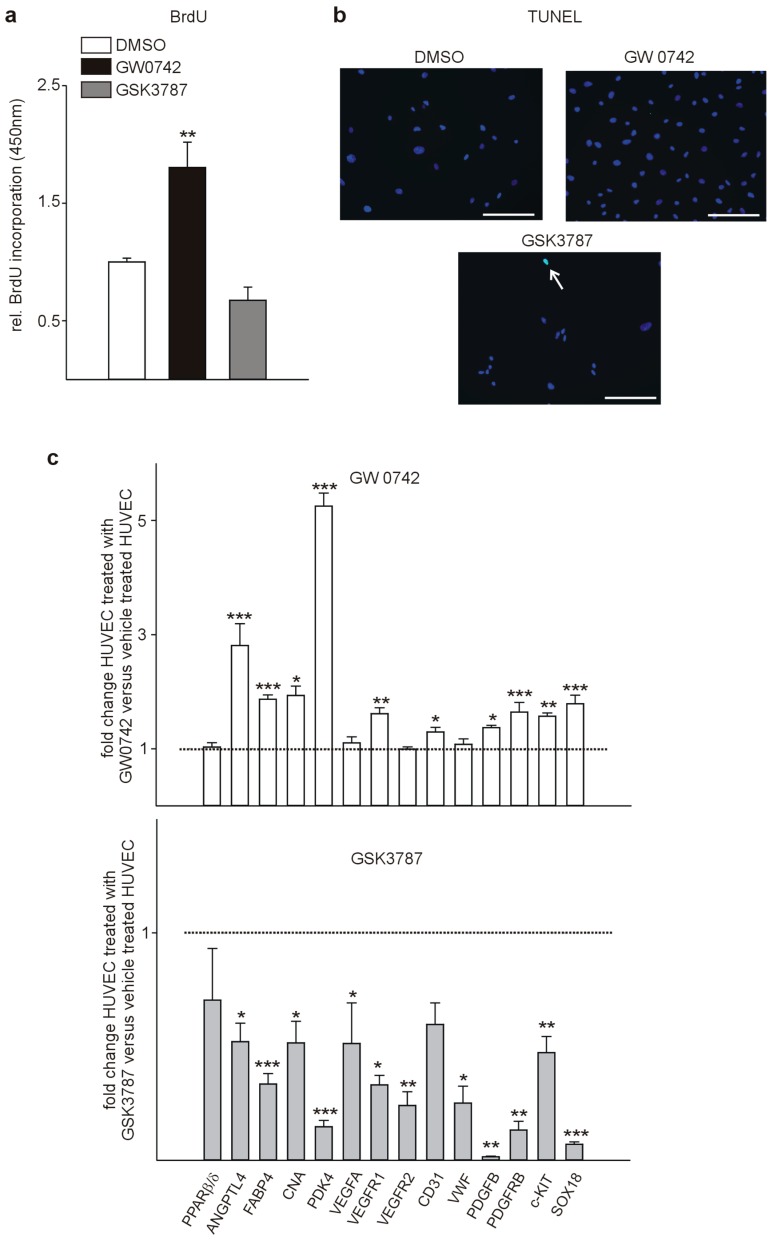
PPARβ/δ agonist GW0742 increases human umbilical vein endothelial cells (HUVEC) proliferation and upregulates expression of angiogenic genes. (**a**) Quantification of BrdU incorporation assays (*n* = 7). (**b**) TUNEL-labeling as a marker for apoptosis (*n* = 3). Note that nearly no TUNEL labeling could be observed independent of the condition. The white arrow points to one of the rare TUNEL positive cells. (**c**) Quantitative RT-PCRs of HUVECs treated with the PPARβ/δ agonist GW0742 and the antagonist GSK3787 (*n* = 6). Scale bars indicate 50 µm. Data are mean ± SEM. ^∗^ *p* < 0.05, ^∗∗^ *p* < 0.01, ^***^ *p* < 0.001.

Treatment of human umbilical vein endothelial cells (HUVECs) with GW0742 increased proliferation, while the PPARβ/δ antagonist GSK3787 tended to reduce HUVEC proliferation (Figure 4a). This is in agreement with previous works reporting enhanced endothelial cell proliferation [34] and activation of angiogenesis upon PPARβ/δ activation [7] and the general view of PPARβ/δ as a pro-angiogenic factor [4]. As an initial study suggested that PPARβ/δ is involved in endothelial cell apoptosis [35], we tested for differences in apoptosis upon modulation of PPARβ/δ. Rarely, TUNEL-positive endothelial cells were observed under any of the culture conditions (Figure 4b), suggesting that PPARβ/δ modulation does not influence apoptosis in vascular cells.

To identify molecular events in the observed pro-angiogenic effect of the PPAR agonist GW0742 on HUVECs, we performed quantitative RT-PCRs. First, we confirmed up-regulation of established PPARβ/δ target genes as Angiopoietin-like 4 (ANGPTL4) [36], Fatty acid binding protein 4 (FABP4) [37], Calcineurin (CNA) [8], and Pyruvate dehydrogenase kinase isoform 4 (PDK4) [38]. Next, we evaluated the expression of genes known to be involved in angiogenesis and found many of them to be up-regulated upon agonist treatment: Vascular endothelial growth factor receptor 1 (Vegfr1), which is critical for endothelial cell survival [39], and platelet/endothelial cell adhesion molecule-1 (CD31), regulating the endothelial cell vascular permeability barrier [40], Platelet-derived growth factor subunit B (PDGFB) and PDGF beta-receptor (PDGFRb), enhancing angiogenesis [17,41], Tyrosinekinase Kit (c-KIT), expressed on endothelial cells [42], implicated in tumor angiogenesis [43], and increasing migration and tube formation of endothelial cells [15,44], and also SRY-related HMG-box transcription factor 18 (SOX18), mediating physiologic and pathologic angiogenesis [45]. Although it has been suggested that Vascular endothelial growth factor (VEGF) is regulated by PPARβ/δ [7,46], we did not observe a statistically significant increase in expression, and no changes in Vascular endothelial growth factor receptor 2 (VEGFR2) expression and Von Willebrand factor (VWF), controlling blood vessel formation [47] (Figure 4c, upper panel). However, in HUVECs treated with the PPARβ/δ antagonist GSK3787, all of the mentioned genes were downregulated (Figure 4c, lower panel) supporting a pro-angiogenic function of PPARβ/δ.

### 3.4. Inducible Vascular-Specific Overexpression of PPARβ/δ Promotes Tumor Angiogenesis, Growth, and Spontaneous Metastases Formation In Vivo

**Figure 5:**
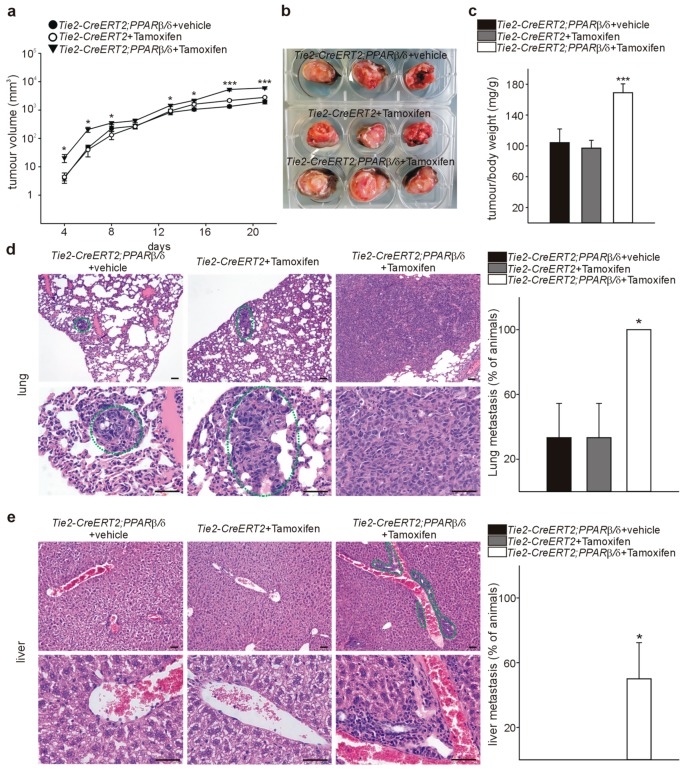
Vascular- specific PPARβ/δ overexpression increases LLC1 tumor growth and spontaneous metastases formation in vivo. (**a**) LLC1 tumor growth curves in Tie2-CreERT2;PPARβ/δ + Tamoxifen animals (*n* = 6) and respective controls (Tie2-CreERT2;PPARβ/δ + vehicle (*n* = 6) and Tie2-CreERT2 + Tamoxifen (*n* = 6). (**b**) LLC1 tumors in Tie2-CreERT2;PPARβ/δ + Tamoxifen mice and control groups. (**c**) Quantification of tumor/body weights. (**d**) Representative photomicrographs of lung metastases in the three groups of mice. Metastases are indicated with a dotted green circle. Note that in Tie2-CreERT2;PPARβ/δ + Tamoxifen animals, metastases were so considerable in size that the whole photomicrograph shows a metastasis only. The plot on the right shows quantification of the percentage of animals with lung metastases. (**e**) Representative photomicrographs of liver sections from the three groups of animals. Metastases are indicated with a dotted green circle. In the two control groups, no liver metastases were detectable. Quantification of the percentage of animals with liver metastases is shown on the right. Scale bars indicate 50 µm. Data are mean ± SEM. ^∗^ *p* < 0.05, ^∗∗∗^*p* < 0.001.

To determine further the functional relevance of PPARβ/δ for tumor vessel formation and tumorigenesis, we used PPARβ/δ-flox^+/−^ mice [12] crossed with Tamoxifen-inducible Tie2-CreERT2 animals. This Cre becomes activated in endothelial cells upon Tamoxifen induction [13]. Using this strategy, we obtained Tie2-CreERT2;PPARβ/δ animals with an inducible conditional vascular-specific overexpression of PPAR*β*/*δ* [10]. In adult Tie2-CreERT2;PPARβ/δ, Cre was activated by tamoxifen injection. Tie2-CreERT2;PPARβ/δ injected with vehicle and Tie2-CreERT2 mice injected with tamoxifen served as controls. We investigated the effects of vascular specific PPARβ/δ overexpression on tumor growth by overexpressing PPARβ/δ in Tie2^+^ cells, followed by subcutaneous implantation of LLC1 tumor cells. Tumor growth curves revealed increased tumor growth rates in mice with vessel- specific PPARβ/δ overexpression (Figure 5a). Three weeks after tumor cell injection, we determined tumor/body weight ratios. Body weights were comparable in all groups of mice (*CreERT2*;PPARβ/δ + vehicle 36.57 g ± 0.44 g, Tie2-CreERT2 + Tamoxifen 38.75 g ± 0.12 g, Tie2-CreERT2;PPARβ/δ + Tamoxifen 36.77 g ± 0.27 g). Of note, tumor weights were nearly doubled in the mice with vascular PPARβ/δ overexpression (Figure 5b,c). Next, we analyzed whether conditional overexpression of PPARβ/δ in vessels might modify metastasis occurrence. In this respect, the LLC1 model is very useful as it forms spontaneous metastases [15]. We investigated serial lung and liver sections of Tie2-CreERT2;PPARβ/δ + Tamoxifen mice and the respective control groups for metastases. All mice with vascular PPARβ/δ overexpression had lung metastases compared to 30% in the control groups (Figure 5d), and 50% displayed liver metastases whereas in the controls no liver metastases formation could be observed (Figure 5e). These observations strengthen the hypothesis that vascular PPARβ/δ enhances tumor growth and spontaneous metastatic spreading; and they are in perfect agreement with tumor growth inhibition and prolonged survival observed in PPARβ/δ^-/-^ mice [33].

**Figure 6:**
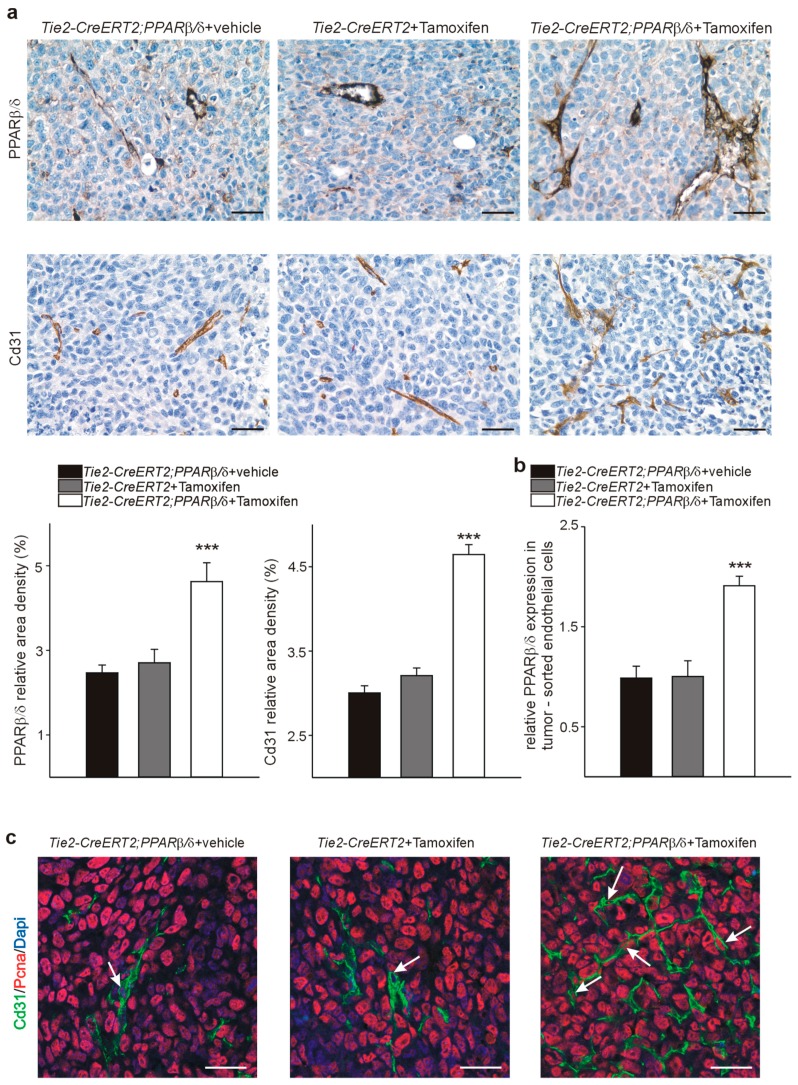
PPARβ/δ and Cd31 are increased in LLC1 tumors from mice with vascular PPARβ/δ overexpression. (**a**) PPARβ/δ (upper panel) and Cd31 (lower panel) immunostainings in LLC1 tumors and quantification of PPARβ/δ and Cd31 signal area densities. (**b**) Quantitative RT-PCRs for PPARβ/δ from tumor sorted endothelial cells of Tie2-CreERT2;PPARβ/δ + Tamoxifen animals and respective controls (*n* = 6 each). (**c**) PCNA/Cd31 double-labeling of LLC1 tumors from Tie2-CreERT2;PPARβ/δ + Tamoxifen animals and respective controls. Scale bars indicate 50 µm. Data are mean ± SEM. ^∗∗∗^ *p* < 0.001.

We analyzed next PPARβ/δ and Cd31 expression in the tumors by immunostaining. Tie2-CreERT2;PPARβ/δ + Tamoxifen tumors displayed much higher PPARβ/δ and Cd31 positive vessel-and area-densities than both control groups (Figure 6a). To confirm the endothelial PPARβ/δ overexpression on the RNA level, we sorted endothelial cells from the tumors and performed quantitative RT-PCRs. PPARβ/δ expression was nearly doubled in the tumor-derived endothelial cells from Tie2-CreERT2;PPARβ/δ + Tamoxifen mice compared to both controls (Figure 6b). Co-immunostaining for PCNA and Cd31 demonstrated more PCNA-positive endothelial cells in the tumors from animals with vessel-specific overexpression of PPARβ/δ than in the controls, indicating higher proliferation rates of endothelial cells, like it had been the case in the tumors of GW0742 agonist treated mice (Figure 6c).

### 3.5. PPARβ/δ Overexpression Upregulates Angiogenic and Metastases Promoting Molecules in Endothelial Cells In Vivo

**Figure 7:**
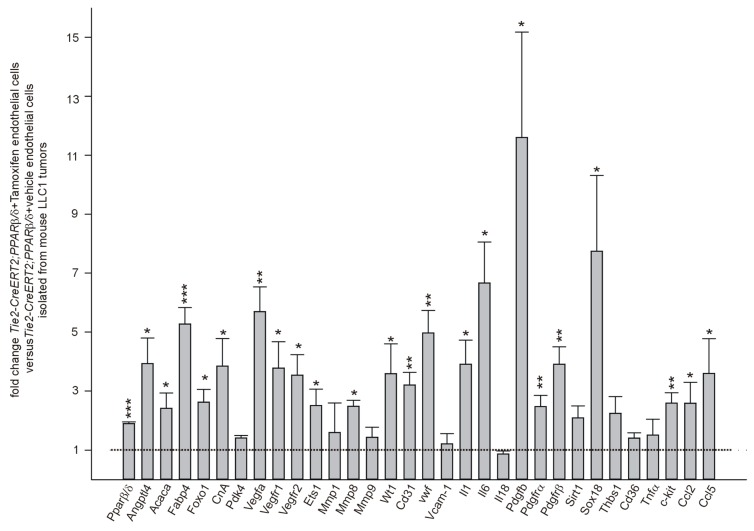
Quantitative RT-PCR corroborates angiogenic functions of PPARβ/δ in endothelial cells. Quantitative RT-PCR of known PPARβ/δ target genes, inflammation, and angiogenesis markers in tumor- sorted endothelial cells from LLC1 bearing Tie2-CreERT2;PPARβ/δ + Tamoxifen animals and Tie2-CreERT2;PPARβ/δ +vehicle mice as controls (*n* = 6 each). Data are mean ± SEM. ^∗^*P* < 0.05, ^∗∗^*P* < 0.01, ^∗∗∗^*P* < 0.001.

To confirm the differential expression of genes we had observed upon pharmacological PPARβ/δ modulation in human endothelial cells and to elucidate further the molecular mechanisms upon PPARβ/δ overexpression in vascular cells in vivo, we performed quantitative RT-PCRs from tumor-sorted endothelial cells (Figure 7). Again, established PPARβ/δ target genes were upregulated except Pdk4, as likewise genes with established angiogenic functions: Vegf, Vegfr1 and 2, Cd31, vwf, Pdgfb, Pdgfrα, and β, Sox18, and c-kit. Furthermore, we found an increase of tumor-angiogenesis related genes such as E26 transformation specific factor 1 (Ets-1) [48,49], and Wt1 (Wilms’ tumor suppressor 1) [15,48]. Interestingly, we could demonstrate that PPARβ/δ represses WT1 in human melanoma cells leading to decreased melanoma cell proliferation [5]. The upregulation of Wt1 upon overexpression of PPARβ/δ in endothelial cells observed here, could be due to cell type-specific expression of co-activators/co-repressors, which requires further studies. We additionally analyzed expression of matrix metalloproteinases 1, 8, and 9. It has been observed that pharmacological activation of PPARβ/δ in vascular cells increased MMP9 mRNA levels [7]. However, we could only find an increase for MMP8, which is known to positively regulate angiogenesis [50] in our model. Interleukin 1 and 6 (Il 1 and Il 6) expression was increased, pointing to an inflammatory state of endothelial cells; yet, Interleukin 18 (Il 18) and Tumor necrosis factor alpha (TNFα) expression were unchanged. Sirt1 and Vcam-1 were not differentially expressed. The mRNA expression of anti-angiogenic Thrombospondin -1 and its receptor Cd36 [51] was unaffected by PPARβ/δ overexpression. It seems that vascular overexpression of PPARβ/δ promotes exclusively a hyper-angiogenic phenotype rather than repressing anti-angiogenic pathways. This might contribute to the observed relatively weak phenotype of PPARβ/δ knockout animals, which are viable, develop normally, are fertile, and display only light growth, skin, adipose tissue, and myelinization alterations [52]. Finally, we observed upregulation of the metastases promoting [53,54] CC-chemokine ligands 2 and 5 (Ccl 2 and 5) in tumor- derived PPARβ/δ overexpressing endothelial cells (Figure 7).

In summary of these findings, the use of PPARβ/δ agonists, initially developed to treat hyperlipidemia or cardiovascular diseases, as anti-cancer drugs, even in the setting of an antiproliferative effect on the tumor cells, seems irresponsible. Although the PPARβ/δ agonist 501,516 entered clinical trials for the treatment of metabolic syndrome and diabetes in the beginning of 2000, these trials were stopped in 2007 due to multiple appearance of cancers in mice and rats (cited in [55]. Unfortunately, upon publication of the beneficial effects of PPARβ/δ activation against obesity and on exercise endurance in mice [12,56,57], the PPARβ/δ agonist GW501516 became very popular in the athletes community.

### 3.6. RNA Sequencing Further Certifies the Acquisition of a Highly angiogenic Endothelial Phenotype Upon PPARβ/δ Overexpression and Identifies Pro-Tumor-Angiogenic Signaling Networks

**Figure 8:**
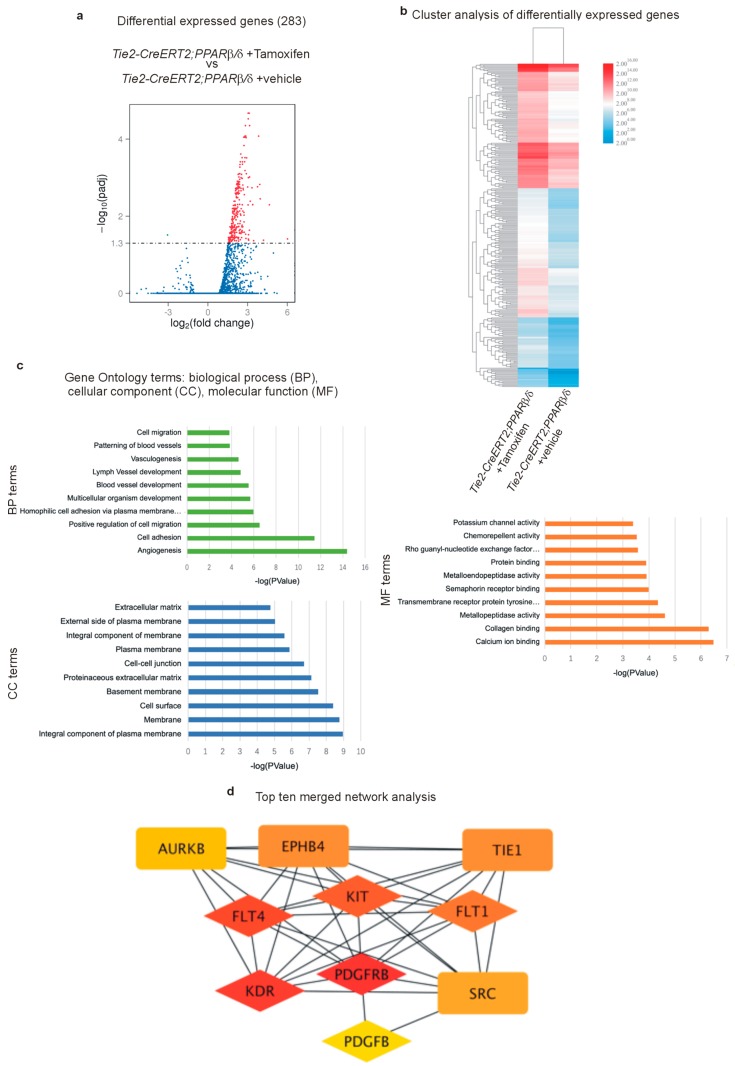
RNA sequencing of tumor- sorted endothelial cells confirms the tumor angiogenesis promoting effect of PPARβ/δ. (**a**) Volcano plot analysis of differentially expressed genes in tumor endothelial cells sorted from LLC1 tumors of Tie2-CreERT2;PPARβ/δ + Tamoxifen animals and Tie2-CreERT2;PPARβ/δ + vehicle mice as controls. (*n* = 5 each). (**b**) Cluster analysis of differentially expressed genes. (**c**) Most significantly changed gene ontology terms (BP: biological process, MF: molecular function, CC: cellular component). (**d**) Top ten network analysis merged with the prediction of PPAR-responsive elements (PPRE): diamonds and rectangles represent the top ten network; but only diamonds exhibited PPRE prediction. As the Vascular endothelial growth factor (VEGF) pathway has been suggested already to be regulated by PPARβ/δ [7,46], we decided to investigate further a potential regulation of Pdgfrβ, Pdgfb, and c-kit.

To analyze the transcriptome of gene expression patterns in PPARβ/δ overexpressing tumor- derived endothelial cells, we sorted endothelial cells from the tumors of Tie2-CreERT2;PPARβ/δ injected with Tamoxifen, or vehicle as controls. mRNAs were after purity and quality controls submitted to sequencing. 283 genes were found to be differentially expressed in vascular cells overexpressing PPARβ/δ, most of them were up-regulated (Figure 8a and Supplementary Table S1). Cluster analysis of differentially expressed genes confirms mostly up-regulation of genes upon vascular overexpression of PPARβ/δ (Figure 8b). Again, this is in line with our observation in the quantitative PCRs, which indicate an angiogenesis boosting effect rather than a repression of anti-angiogenic molecules to enhance angiogenesis. Interestingly, gene ontology term analysis identified angiogenesis as the most upregulated biological process upon PPARβ/δ overexpression, followed by cell adhesion (Figure 8c). Top ten network analysis combined with a search for PPAR responsive elements (PPREs) identified in total six genes with potential PPREs: the three Vegf receptors 1 (Flt1), 2 (Kdr), and 3 (Flt4), all of them known to be implicated in the promotion of tumor angiogenesis [58,59], and Pdgfrβ, Pdgfb, and c-kit, which we had already found to be upregulated upon PPARβ/δ overexpression (Figure 7). Initial clinical efforts to inhibit tumor angiogenesis mainly focused on inhibition of the VEGF/VEGFR signaling, but often further disease progression could be observed. Angiogenesis involves multiple pathways, and it became clear that inhibition of only one pathway could be compensated by the others resulting in tumor progression. One of these compensatory pathways is platelet-derived growth factor PDGF and PDGFR signaling which led to the development of new anti-angiogenic therapies in the treatment of cancer [60]. Additionally, c-Kit belongs to the group of tumor angiogenesis-promoting molecules and new tyrosine kinase receptor inhibitors have been developed which target also c-Kit [61].

### 3.7. PPARβ/δ Directly Activates PDGFRβ, PDGFB, and c-Kit

**Figure 9:**
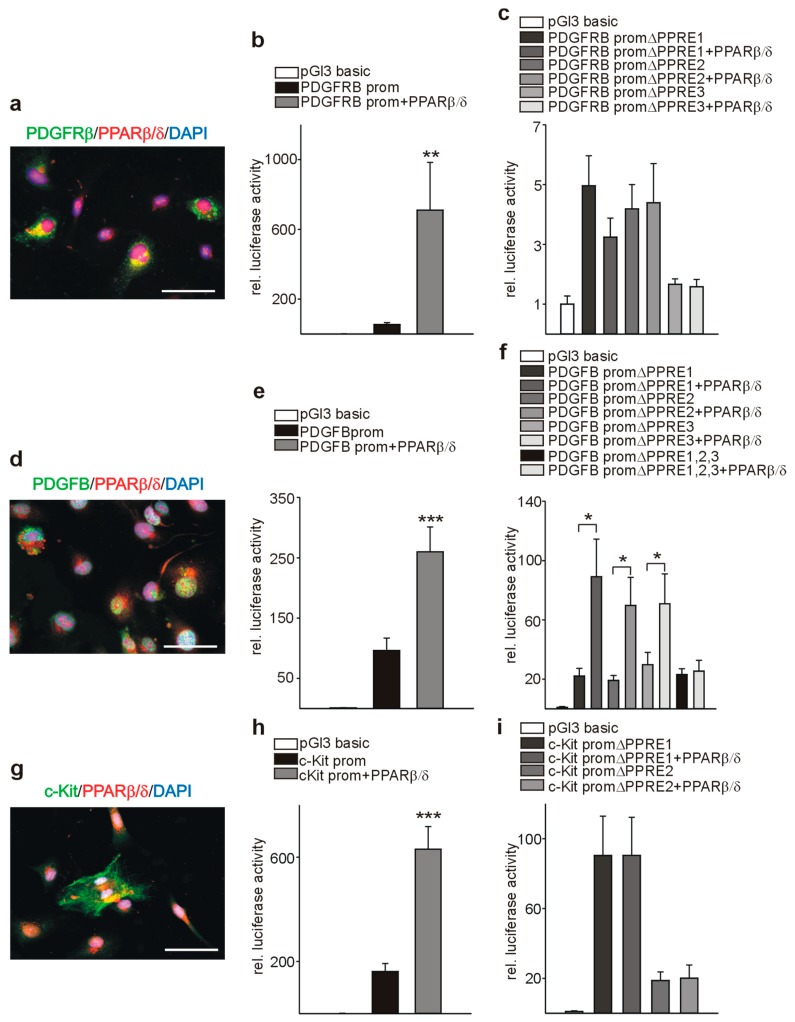
PPARβ/δ transactivates PDGFRb, PDGFB, and c-Kit promoters. (**a**) Co-labeling for PPARβ/δ (red) and PDGFRβ (green) in endothelial cells. (**b**) Luciferase activity of reporter constructs carrying the PDGFRβ promoter in the presence of the PPARβ/δ expression construct (*n* = 12 each). (**c**) Transient transfections of luciferase constructs carrying the PDGFRβ promoter with deletion of the identified PPREs (ΔPPRE) in the presence of the PPARβ/δ expression construct (*n* = 12 each). (**d**) Co-immunostaining for PPARβ/δ (red) and PDGFB (green) in endothelial cells. (**e**) Luciferase activity of reporter constructs carrying the PDGFB promoter in the presence of the PPARβ/δ expression construct (*n* = 12 each). (**f**) Transient transfections of luciferase constructs carrying the PDGFB promoter with deletion of the identified PPREs (ΔPPRE) in the presence of the PPARβ/δ expression construct (*n* =16 each). (**g**) Co-labeling for PPARβ/δ (red) and c-Kit (green) in endothelial cells. (**h**) Transient transfections of luciferase reporter constructs carrying the c-Kit promoter in the presence of the PPARβ/δ expression construct (*n* = 10 each). (**i**) Luciferase activity of constructs carrying the c-Kit promoter with deletion of the identified PPREs (ΔPPRE) in the presence of the PPARβ/δ expression construct (*n* = 6 each). The promoter-less luciferase expression construct (pGl3basic) served as a negative control in all transfection experiments. Luciferase activities were normalized for the activity of co-transfected β-galactosidase. Scale bars indicate 50 µm. Data are mean ± SEM. ^∗^*P* < 0.05, ^∗∗^*P* < 0.01, ^∗∗∗^*P* < 0.001.

**Figure 10:**
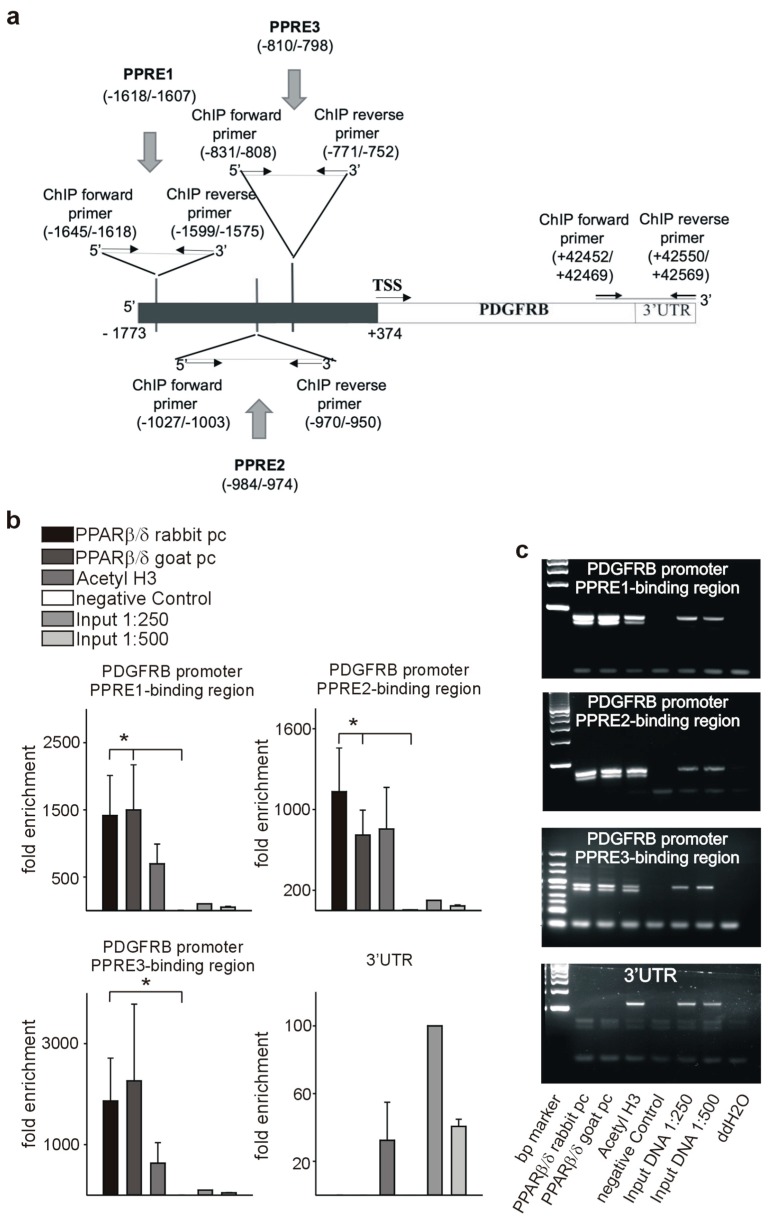
PPARβ/δ binds to the PDGFRβ promoter. (**a**) Schematic representation of the putative PPREs in the PDGFRβ promoter. Positions of the cloned promoters relative to the transcription start site, positions and sequences of the putative PPREs, and positions of the oligonucleotides used for CHIP analyses are indicated. For promoter-deletion constructs, the indicated PPREs were removed from the promoter reporter constructs. Chromatin immunoprecipitation (ChIP, *n* = 4) was performed using polyclonal antibodies against PPARβ/δ or anti-acetyl-histone H3 antibody. Input DNA was used as a positive control for quantitative PCRs (**b**) or semiquantitative PCRs ((**c**), representative agarose gels) for the PDGFRβ promoter and respective 3′UTR sequences. Data are mean ± SEM. ∗*p* < 0.05.

**Figure 11:**
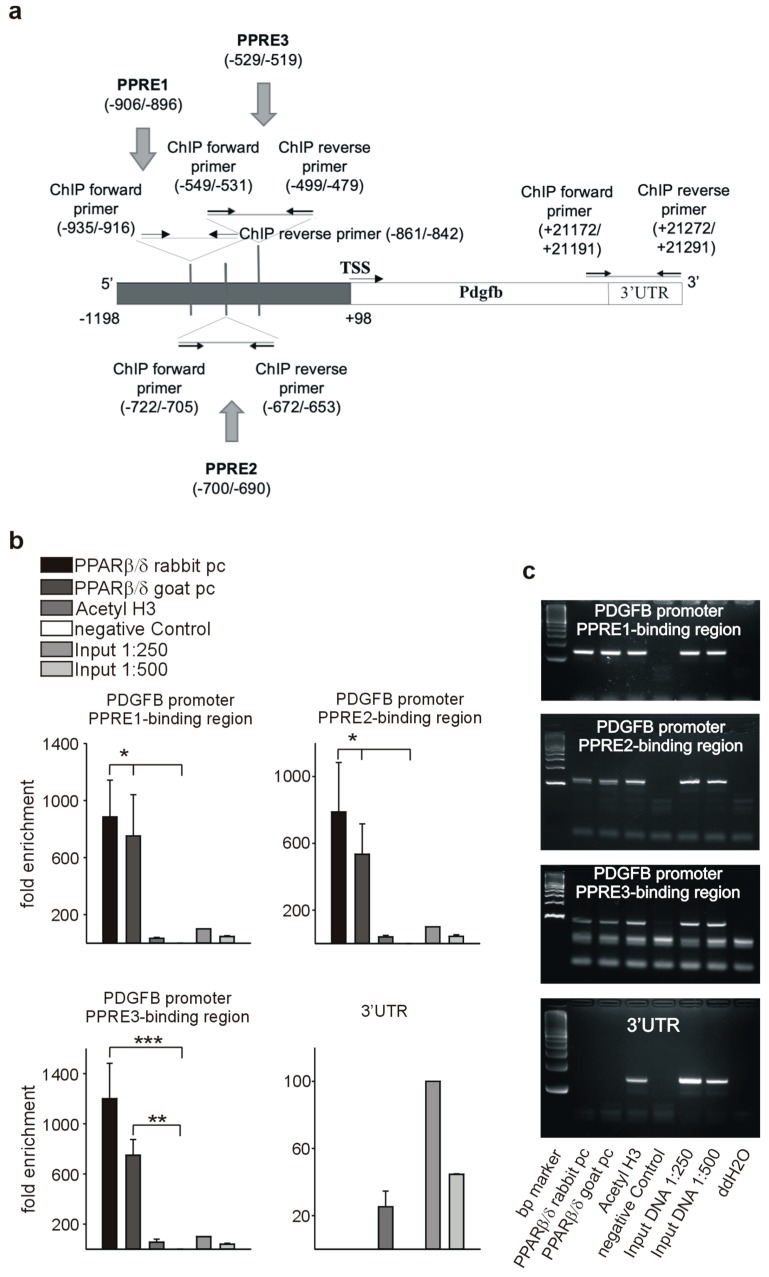
PPARβ/δ binds to the PDGFB promoter. (**a**) Schematic representation of the putative PPREs in the PDGFB promoter. Positions of the cloned promoters relative to the transcription start site, positions and sequences of the putative PPREs, and positions of the oligonucleotides used for CHIP analyses are indicated. For promoter-deletion constructs, the indicated PPREs were removed from the promoter reporter constructs. Chromatin immunoprecipitation (ChIP, *n* = 3) was performed using polyclonal antibodies against PPARβ/δ or anti-acetyl-histone H3 antibody. Input DNA was used as a positive control for quantitative PCRs (**b**) or semiquantitative PCRs ((**c**), representative agarose gels) for the PDGFRβ promoter and respective 3′UTR sequences. Data are mean ± SEM. ^∗^*p* < 0.05, ^∗∗^*p* < 0.01.

**Figure 12:**
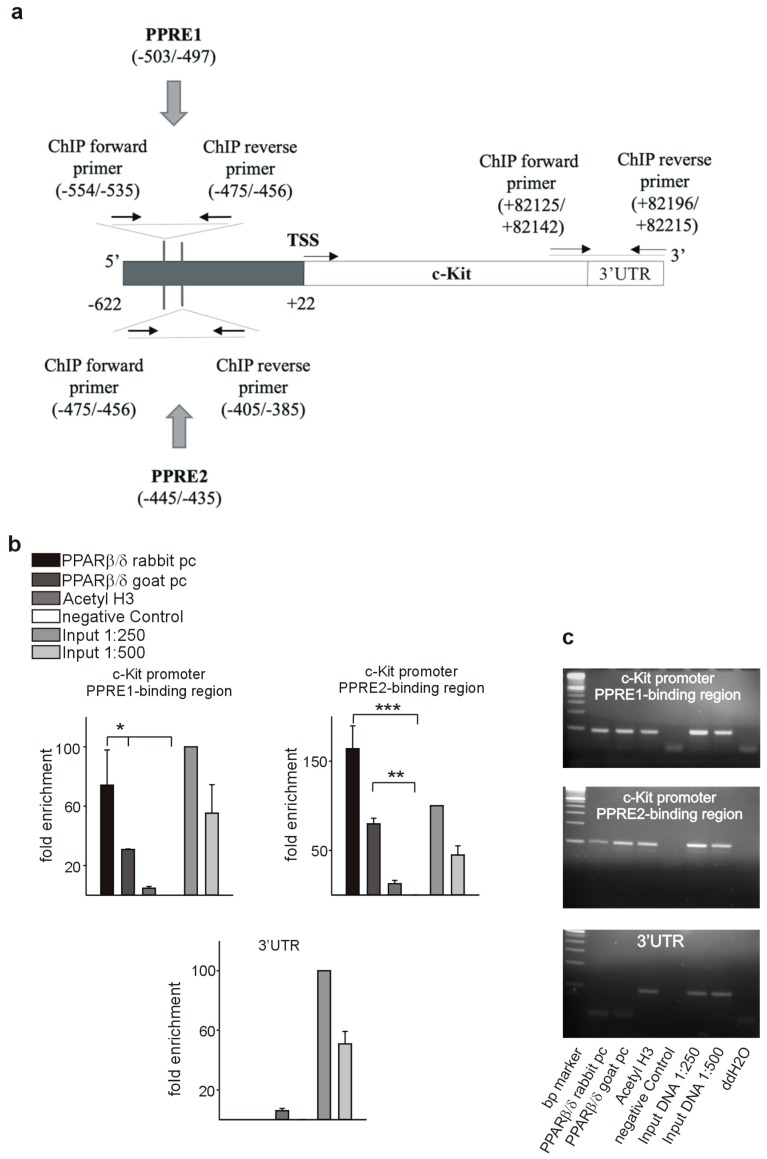
PPARβ/δ binds to the c-Kit promoter. (**a**) Schematic representation of the putative PPREs in the c-Kit promoter. Positions of the cloned promoters relative to the transcription start site, positions and sequences of the putative PPREs, and positions of the oligonucleotides used for CHIP analyses are indicated. For promoter-deletion constructs, the indicated PPREs were excluded from the promoter reporter constructs. Chromatin immunoprecipitation (ChIP, *n* = 3) was performed using polyclonal antibodies against PPARβ/δ or anti-acetyl-histone H3 antibody. Input DNA was used as a positive control for quantitative PCRs (**b**) or semiquantitative PCRs ((**c**), representative agarose gels) for the PDGFRβ promoter and respective 3′UTR sequences. Data are mean ± SEM. ^∗^*p* < 0.05, ^∗∗^*p* < 0.01, ^∗∗∗^*p* < 0.001.

As a prerequisite for transcriptional regulation, we first demonstrated co-localization of PPARβ/δ and PDGFRβ (Figure 9a), PDGFB (Figure 9d), and c-Kit (Figure 9g) in endothelial cells. Transient co-transfection with a PPARβ/δ expression construct increased the activities of PDGFRβ (Figure 9b), PDGFB (Figure 9e), and c-Kit (Figure 9h) promoter reporter constructs. Using in silico analysis, PPAR responsive elements (PPREs) were identified in the respective promoter sequences (Figure 10a, Figure 11a, Figure 12a). Deletion of each of the three identified PPREs in the PDGFRβ promoter was sufficient to abolish transactivation by PPARβ/δ (Figure 9c). Regarding the PDGFB promoter, only the combined deletion of the three identified PPREs abrogated activation upon co-transfection of the PPARβ/δ construct, indicating a collaborative interaction between these PPREs (Figure 9f). Finally, in the c-Kit promoter, each deletion for the two identified binding regions was sufficient to cancel activation by PPARβ/δ (Figure 9i).

Binding of PPARβ/δ to the predicted PPREs was confirmed by chromatin immunoprecipitation (ChIP) assays. An antibody against acetylated histone H3 was used to check for nucleosome integrity. Specificity of the interaction of PPARβ/δ with the PPAR responsive elements in the PDGFRβ promoter is indicated by the lack of a PCR product when the same samples were amplified with primers specific for the 3′ UTR. We confirmed binding to the identified PPREs in the PDGFRβ (Figure 10b,c), PDGFB (Figure 11b,c), and the c-Kit promoter (Figure 12b,c).

## 4. Conclusions

We show here that pharmacological PPARβ/δ activation increases endothelial cell proliferation in vitro and intensifies tumor angiogenesis in vivo, enhancing tumor progression and metastases formation. Conditional vascular specific overexpression of PPARβ/δ also resulted in enhanced tumor angiogenesis, growth, and metastases formation, suggesting an endothelial cell- specific mechanism for PPARβ/δ function in tumor progression, independent from its contrasting effects on specific tumor cell types.

Tumor-angiogenesis promoting effects of PPARβ/δ are mediated via activation of the PDGF/PDGFR pathway, c-Kit, and probably the VEGF/VEGFR pathway. The pharmacological use of PPARβ/δ agonists should be considered as dangerous regarding its consistent consequences on tumor angiogenesis. Consideration of PPARβ/δ antagonists for inhibition of tumor angiogenesis and cancer growth is unlikely to represent a novel direction due to their controversial effects on the proliferation of different tumor cell types.

## Figures and Tables

**Table 1:** Primer sequences for mouse genes.

Name	Accession Number	Primer Sequences	Amplicon Size (bp) ^1^
PPARβ/δ	NM_011145.3	F: ATGGGGGACCAGAACACAC R: GGAGGAATTCTGGGAGAGGT	62
Angptl4	NM_020581.2	F: CACCCACTTACACAGGCCG R: GAAGTCCACAGAGCCGTTCA	178
Acaca	NM_133360.2	F: GCCTTTCACATGAGATCCAGC R: CTGCAATACCATTGTTGGCGA	175
Fabp4	NM_024406.3	F: TGAAATCACCGCAGACGACA R: ACACATTCCACCACCAGCTT	141
Foxo1	NM_019739.3	F: CAAGGCCATCGAGAGCTCAG R: AATTGAATTCTTCCAGCCCGCC	130
Cna	NM_008913.5	F: AAAGCGCTACTGTTGAGGCT R: ATTCGGTCTAAGCCCTTGGC	103
Pdk4	NM_013743.2	F: TTCCAGGCCAACCAATCCAC R: TGGCCCTCATGGCATTCTTG	87
Vegf	NM_001025250.3	F: CTCACCAAAGCCAGCACATA R: AATGCTTTCTCCGCTCTGAA	198
Vegfr1	NM_010228.4	F: TACCTCACCGTGCAAGGAAC R: AAGGAGCCAAAAGAGGGTCG	93
Vegfr2	NM_010612.3	F: AGTGGTACTGGCAGCTAGAAG R: ACAAGCATACGGGCTTGTTT	65
Ets1	NM_011808.3	F: CTGACCTCAACAAGGACAAGC R: AGAAACTGCCACAGCTGGAT	88
Mmp1	NM_008607.2	F: GGCCAGAACTTCCCAACCAT R: AGCCCAGAATTTTCTCCCTCT	89
Mmp8	NM_008611.4	F: CCTGCAGGACTCCTTCTTCCT R: CCTCATAGGGTGCGTGCAA	156
Mmp9	NM_013599.4	F: CCATGCACTGGGCTTAGATCA R: GGCCTTGGGTCAGGCTTAGA	147
Wt1	NM_144783.2	F: CCAGCTCAGTGAAATGGACA R: CTGTACTGGGCACCACAGAG	97
Cd31	NM_008816.3	F: CGGTGTTCAGCGAGATCC R: CGACAGGATGGAAATCACAA	71
Vwf	NM_011708.4	F: TGTGACACATGTGAGGAGCC R: CTTTGCTGGCACACTTTCCC	127
Vcam1	NM_011693.3	F: TATGTCAACGTTGCCCCCAA R: CAGGACTGCCCTCCTCTAGT	73
Il-1	NM_008361.4	F: GCCACCTTTTGACAGTGATGAG R: AGCTTCTCCACAGCCACAAT	186
Il-6	NM_031168.2	F: CACTTCACAAGTCGGAGGCT R: TGCCATTGCACAACTCTTTTCT	86
Il-18	NM_008360.2	F: CAAAGTGCCAGTGAACCCCA R: TTCACAGAGAGGGTCACAGC	89
Pdgfb	NM_011057.4	F: GGAGTCGGCATGAATCGCT R: GCCCCATCTTCATCTACGGA	182
Pdgfra	NM_001083316.2	F: ATGAGAGTGAGATCGAAGGCA R: CGGCAAGGTATGATGGCAGAG	130
Pdgfrb	NM_001146268.1	F: CCAGCACCTTTGTTCTGACCT R: TGCCGTCCTGATTCATGGC	99
Sirt1	NM_019812.3	F: GCCGCGGATAGGTCCATA R: AACAATCTGCCACAGCGTCA	136
Sox18	NM_009236.2	F: ACTGGCGCAACAAAATCC R: CTTCTCCGCCGTGTTCAG	88
Thbs1	NM_011580.4	F: CCTGCCAGGGAAGCAACAA R: ACAGTCTATGTAGAGTTGAGCCC	115
Cd36	NM_001159555.1	F: GTGTGGAGCAACTGGTGGAT R: ACGTGGCCCGGTTCTAATTC	147
Tnfα	NM_013693.3	F: GTAGCCCACGTCGTAGCAAA R: ACAAGGTACAACCCATCGGC	137
c-kit	NM_001122733.1	F: GCCTGACGTGCATTGATCC R: AGTGGCCTCGGCTTTTTCC	110
Ccl2	NM_011333.3	F: AGCTGTAGTTTTTGTCACCAAGC R: GTGCTGAAGACCTTAGGGCA	155
Ccl5	NM_013653.3	F: TGCAGTCGTGTTTGTCACTC R: AGAGCAAGCAATGACAGGGA	152
Actnb	NM_007393.5	F: CTTCCTCCCTGGAGAAGAGC R: ATGCCACAGGATTCCATACC	124
Gapdh	NM_001289726.1	F: AGGTCGGTGTGAACGGATTTG R: TGTAGACCATGTAGTTGAGGTCA	123
Rplp0	NM_007475.5	F: CACTGGTCTAGGACCCGAGAAG R: GGTGCCTCTGGAGATTTTCG	73

**^1^** bp: base pairs; F: forward primer sequence; R: reverse primer sequence.

**Table 2:** Primer sequences for human genes.

Name	Accession Number	Primer Sequences	Amplicon Size (bp) ^1^
PPARβ/δ	NM_006238.5	F: TCAGAAGAAGAACCGCAACAAGTG R: CCTGCCACCAGCTTCCTCTT	126
ANGPTL4	NM_001039667.3	F: ATCCAGCAACTCTTCCACAAGGT R: TTGAAGTCCACTGAGCCATCGT	254
FABP4	NM_001442.3	F: AAGTCAAGAGCACCATAACCTTAGATG R: TGACGCATTCCACCACCAGTT	120
CNA	NM_000944.5	F: AAAGCGCTACTGTTGAGGCT R: ATTCGGTCTAAGCCCTTGGC	103
PDK4	NM_002612.4	F: CCACATTGGAAGCATTGATCCTAACT R: TCACAGAGCATCCTTGAACACTCA	81
VEGFA	NM_001025366.3	F: GAGGAGTCCAACATCACCATGC R: CTTGCAACGCGAGTCTGTGTT	351
VEGFR1	NM_002019.4	F: GCCCGGGATATTTATAAGAAC R: CCATCCATTTTAGGGGAAGTC	70
VEGFR2	NM_002253.3	F: CAGAGTGAGGAAGGAGGACGAAGG R: GATGATGACAAGAAGTAGCCAGAAGAACA	181
CD31	NM_000442.5	F: GCCCGAAGGCAGAACTAAC R: AACAGAGCAGAAGGGTCAG	111
VWF	NM_000552.4	F: TGTGACACATGTGAGGAGCC R: CTTTGCTGGCACACTTTCCC	127
PDGFB	NM_002608.4	F: TCCGCTCCTTTGATGATCTCCAA R: GGTCATGTTCAGGTCCAACTCG	83
c-KIT	NM_000222.2	F: GCTCTGCTTCTGTACTGCC R: TAGGCAGAAGTCTTGCCCAC	160
SOX18	NM_018419.3	F: ATGGTGTGGGCAAAGGAC R: GCGTTCAGCTCCTTCCAC	107
ACTNB	NM_001101.5	F: CTCCTTAATGTCACGCACGAT R: CATGTACGTTGCTATCCAGGC	250
GAPDH	NM_002046.7	F: AGCCACATCGCTCAGACAC R: GCCCAATACGACCAAATCC	66
RPLP0	NM_001002.4	F: CAGATTGGCTACCCAACTGTT R: GGCCAGGACTCGTTTGTACC	69

**^1^** bp: base pairs; F: forward primer sequence; R: reverse primer sequence.

## Supplementary Materials

The following is available online at https://www.mdpi.com/2073-4409/8/12/1623/s1, Table S1.
